# Reflectance Measurement Method Based on Sensor Fusion of Frame-Based Hyperspectral Imager and Time-of-Flight Depth Camera

**DOI:** 10.3390/s22228668

**Published:** 2022-11-10

**Authors:** Samuli Rahkonen, Leevi Lind, Anna-Maria Raita-Hakola, Sampsa Kiiskinen, Ilkka Pölönen

**Affiliations:** Faculty of Information Technology, University of Jyväskylä, 40014 Jyväskylä, Finland

**Keywords:** hyperspectral, depth data, kinect, sensor fusion, reflectance

## Abstract

Hyperspectral imaging and distance data have previously been used in aerial, forestry, agricultural, and medical imaging applications. Extracting meaningful information from a combination of different imaging modalities is difficult, as the image sensor fusion requires knowing the optical properties of the sensors, selecting the right optics and finding the sensors’ mutual reference frame through calibration. In this research we demonstrate a method for fusing data from Fabry–Perot interferometer hyperspectral camera and a Kinect V2 time-of-flight depth sensing camera. We created an experimental application to demonstrate utilizing the depth augmented hyperspectral data to measure emission angle dependent reflectance from a multi-view inferred point cloud. We determined the intrinsic and extrinsic camera parameters through calibration, used global and local registration algorithms to combine point clouds from different viewpoints, created a dense point cloud and determined the angle dependent reflectances from it. The method could successfully combine the 3D point cloud data and hyperspectral data from different viewpoints of a reference colorchecker board. The point cloud registrations gained 0.29–0.36 fitness for inlier point correspondences and RMSE was approx. 2, which refers a quite reliable registration result. The RMSE of the measured reflectances between the front view and side views of the targets varied between 0.01 and 0.05 on average and the spectral angle between 1.5 and 3.2 degrees. The results suggest that changing emission angle has very small effect on the surface reflectance intensity and spectrum shapes, which was expected with the used colorchecker.

## 1. Introduction

Extracting meaningful information from a combination of different imaging modalities, such as standard RGB images, hyperspectral data and depth maps produced by depth perceiving cameras, is a demanding task. Fusing data of different types, volumes and dimensions from varying sources and different sensors is a research area with a lot of emerging new technologies and applications.

Image sensor fusion requires knowing the optical properties of the sensors, selecting the right optics and the finding sensors’ mutual reference frame through calibration. In our case, producing a hyperspectral point cloud also requires estimating the relative positions and orientations of the cameras in the world by using registration algorithms.

Hyperspectral imaging (HSI) considers capturing images with specialized hyperspectral cameras. Each image pixel captures a spectrum of light and each wavelength is captured with a narrow bandwidth. The spectral and spatial dimensions together can be used to characterize and identify points of interest in the image [1].

Previously, depth sensing imaging technologies have shown their ability to add meaningful information to improve, e.g., classification [2], robot navigation [3], and segmenting regions of interest from images [3,4]. In this research, we are using Kinect V2 depth camera. Depth sensing cameras are able to capture depth maps where each pixel corresponds to a distance.

We used Piezo-actuated metallic mirror Fabry–Pérot interferometer (FPI) hyperspectral camera, which is a frame-based imager, developed at the Technical Research Centre of Finland Ltd (VTT) [5]. It captures the scene by taking multiple frames and combining them into a hyperspectral data cube with spatial and spectral dimensions. Each produced pixel in the image corresponds to a mixed radiance spectrum of light, ranging from visible light to infrared wavelengths, depending on the application and the camera. A common quantity measured with these devices is the spectral reflectance of a material, defined as the ratio of reflected and incident light per measured wavelength band.

Frame-based hyperspectral cameras produce an image from a static target without moving the camera itself, as opposed to the whisk broom or push broom type of scanners [6]. Using a frame-based imager makes it easier to fuse the sensor data to other similar imaging modalities.

Hyperspectral imaging has been used in many fields. It can be used non-destructively to conserve, preserve and research objects of our cultural heritage, such as art and historical artifacts [6,7,8]. Many applications apply depth information to hyperspectral images in long range imaging, such as in aerial imaging in forestry [9] and agricultural applications. At close proximity, depth data of complex surfaces can be inferred through controlled illumination of the target and photometric stereo imaging. Skin cancer diagnosis is one medical imaging application of this setup employing a hyperspectral camera [10].

Depth imaging cameras have been used in the past to assist in segmenting objects from the background. Adding depth to hyperspectral images could benefit, e.g., in industrial robot applications where the robot has to gather information, detect and plan actions autonomously based on the sensory input. Example applications could be found for perishable products, such as in automatic fruit inventory and harvesting robots [11]. Hyperspectral imaging has previously been applied for detecting injuries in fruits [12] and with other horticultural products [13].

Combining 3D data from a Kinect V2 with hyperspectral images has previously been done in [14]. The aim of the study was to improve the accuracy of reflectance measurements for curved leaf surfaces by selecting a white reference measurement with the same height and surface normal direction as the sample. This was done by building a white reference library from measurements of a specially designed white reference sample, imaged with the same setup as the leaves.

In [15], the authors developed a 3D multiview RGB-D image-reconstruction method for imaging chlorophyl contents of tomato plants using a multispectral imager and Kinect V2. The used hyperspectral camera employed an internal scanning mechanism where the sensor is moved behind the optics. A plant was rotated around its axis while a Kinect V2 and a hyperspectral camera captured depth images and multispectral images with four selected wavelength bands. The data were used in analyzing spectral reflectance variability from different view angles and to create chlorophyl contents prediction model. The findings suggest that multiview point cloud model could produce superior plant chlorophyl measurements compared to a single-view point cloud model. The camera sensor fusion was carried out by an image registration technique based on Fourier transform, phase correlation and a rotating electric turntable with visible sticker markers.

This research demonstrates a method for fusing frame-based hyperspectral camera data with 3D depth data and an experimental application on how the depth augmented hyperspectral data can be used for measuring angle-wise reflectance of a color checker board. Comparing to the previous linescanner method described in [14], a frame-based imager imposes many benefits in terms of the ease of imaging and portability; setting up the system and capturing a scene does not require a moving linescanner. In our experiment, we selected fitting optics and the calibration method considers common reference points in calibration images and not the spectral domain, such as in the method proposed by [15]. Our imager captured hyperspectral data cubes with 133 wavelength bands. We combined them with the estimated 3D surface normals of the target object and calculated the emission angles.

Novelty of the study come from the camera fusion method of these types of cameras. The findings, challenges and topics on how this kind of data could be utilized in future research will be discussed. This kind of setup could potentially be used in, for example, imaging and researching complex surfaces for material characterization, as well as in specular reflection removal from spectra. In summary, this method provides technical support for designing and implementing a system for hyperspectral 3D point cloud creation and analysis.

## 2. Materials and Methods

### 2.1. Experimental Setup

**Figure 1:**
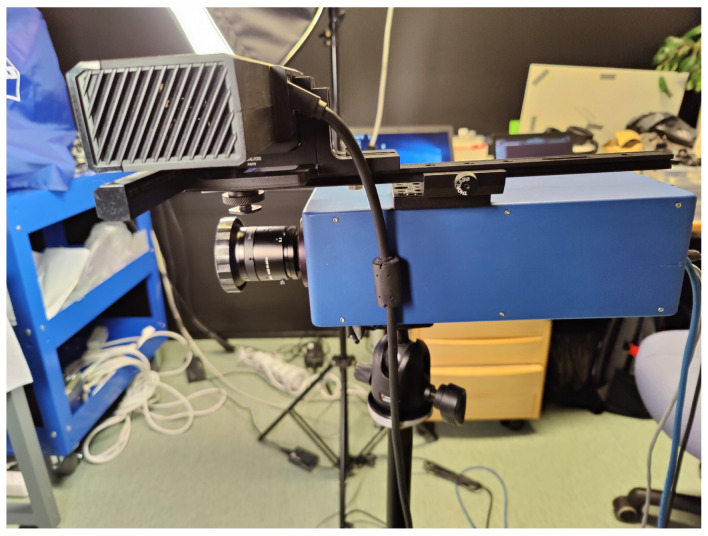
The prototype FPI hyperspectral camera (**below**) and the Kinect (**on top**) used in the research.

The experimental setup consisted of a Fabry-Pérot interferometer (FPI) hyperspectral camera, Microsoft Kinect V2 depth sensing camera, two halogen lights equipped with diffusers, x-rite ColorChecker calibration board and a desk in a darkened room in Spectral imaging laboratory at University of Jyväskylä. The Kinect was aligned on top of the hyperspectral camera and attached and aligned using an assembly of a base, translation rail and mounting brackets by Thorlabs, as seen in Figure 1.

The experimental software for this study was written in Python 3.8 with OpenCV computer vision, Open3D point cloud processing, and Numpy numerical libraries. The software was targeted to work on Ubuntu Linux 20.04 LTS.

### 2.2. FPI Hyperspectral Camera

We used Fabry–Pérot interferometer (FPI) hyperspectral camera developed by VTT Research Centre of Finland. The camera is an assembly of optics, an interferometer, filters, and a machine vision sensor (Grasshopper3 USB3 GS3-U3-23S6C-C) with an RGB sensor. It captures a hyperspectral data cube that has (x, y) spatial dimensions and a spectral domain. The camera works by capturing multiple images and varying the interferometer settings between exposures. The Piezo-actuated interferometer consists of two metallic half-mirrors whose separation can be controlled. A beam of light entering the system interferes with itself as it reflects off the mirrors. Only integer multiples of certain wavelengths get transmitted through the mirrors [16,17].

The hyperspectral camera uses high and low-pass filters to block the unwanted wavelengths of light. Our setup used 450 nm high-pass and 850 low-pass filters, and it was calibrated to capture 80 raw bands from the calibrated 450–850 nm range. The spectral resolution (full width half maximum, FWHM) varied from 8 to 25 nm. We used CubeView [18] software to capture hyperspectral data cubes. The software converted the raw bands to 133 radiance bands using fpipy [19] Python library. The hyperspectral data were stored in fpipy defined netcdf file format with 1920 × 1200 resolution. The file size of one data cube was approximately 4.9 GB.

We aimed to capture sharp and evenly exposed images. Therefore, the aperture was set small (f/8) to have a large depth of field and to minimize vignetting that would otherwise show as a reduction in brightness towards the periphery of the image [20]. The exposure time was set to 3 s per frame to counter small aperture size and underexposed images. The total exposure time for the 80 frames of one hyperspectral image was then approximately 4 min.

### 2.3. Kinect V2

We used Microsoft Kinect V2 depth sensing camera for capturing depth maps of the target. Kinect V2 works by illuminating the scene with infrared light and estimates the distance to obstacles by time-of-flight (TOF) principle. The distance to obstacles is estimated measuring the time it takes light to travel from the emitter back to the infrared camera [21].

We used Libfreenect2 [22] open source driver and a modified Python wrapper based on [23]. The depth maps were captured with 512 × 424 resolution.

### 2.4. Cameras and Optics

**Figure 2:**
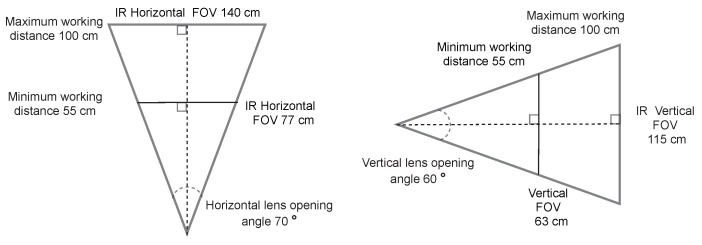
The horizontal and vertical field of view were calculated based on the Kinect V2’s lens opening angles, using 100 cm as a reference maximum working distance for the imaging setup.

Hyperspectral camera optics were selected to provide a similar field of view (FOV) to the Kinect V2, using a 100 cm working distance (WD). Since Kinect’s horizontal and vertical lens opening angles were 70 and 60 degrees, the horizontal and vertical FOV was calculated as seen in Figure 2.

**Figure 3:**
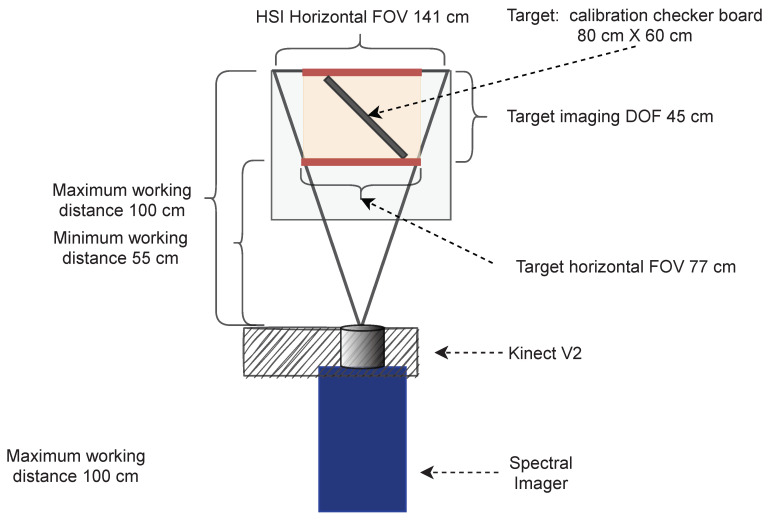
Selecting optics for the target scene. A visualization of the imaging setup parameters for achieving a similar vertical and horizontal field of view with HSI and Kinect V2 sensors.

The defined the required depth of field (DOF) of the target to be 45 cm (Figure 3), which determined the maximum and minimum WDs to be 100 cm and 55 cm, respectively. The resulting horizontal FOV at the minimum WD was 77 cm and 141 cm at the maximum WD.

**Figure 4:**
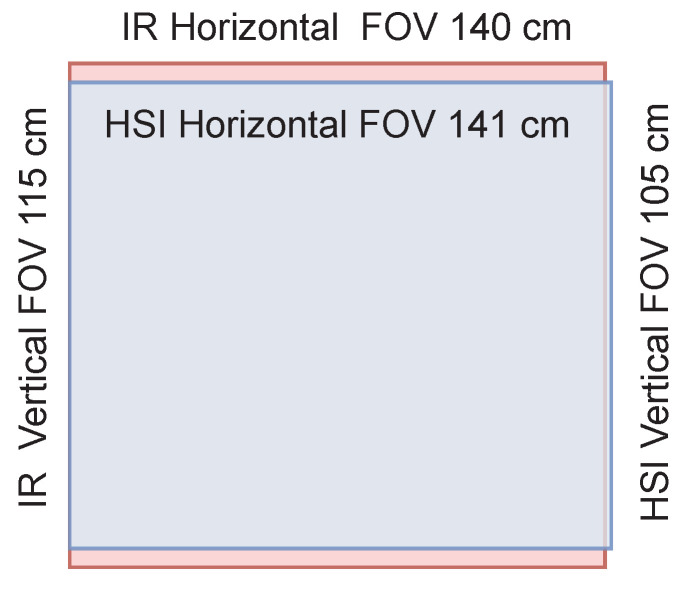
The hyperspectral camera lens provided a relatively similar vertical and horizontal field of views (visualized in blue) than the Kinect V2, which is visualized using red color.

The selected lens was Basler Standard Lens (C10-0814-2M-S f8mm) with C-mount. The fixed focal length was 8.0 mm, and the resolution was 2 megapixels. With hyperspectral camera sensor, the lens provided 141×105 cm FOV, which is visualized with Kinect’s FOV (140×115) cm in Figure 4.

By placing the Kinect on top of the hyperspectral camera and adjusting the lenses’ outer surfaces to the vertically same level, we could capture hyperspectral and depth data with relatively similar parameters (Figure 3 and Figure 4). The HSI sensor was smaller than an ideal sensor for the selected lens, but the possible vignetting effect was controlled by adjusting the iris during the acquisition.

### 2.5. Spectral Point Cloud Generation

In order to combine the depth data and the spectral data from Kinect and the hyperspectral camera, we need to know intrinsic camera parameters and the extrinsic camera parameters. They define the optical properties of the cameras, their relative positions and orientations to each other in the world. The data fusion of the two cameras was carried out as follows: We estimated the global point coordinates seen by Kinect, transform them to the viewpoint of the hyperspectral camera and project them onto its camera plane. Then we match the projected points to the pixels on the hyperspectral camera plane to form the spectral point cloud.

The definition of the camera matrix (also known as the camera intrinsic matrix) for both Kinect and FPI hyperspectral camera is in Equation (1), where $f_{x}$ and $f_{y}$ are the focal lengths in *x* and *y* directions. Correspondingly, $c_{x}$ and $c_{y}$ denote the principal point, which means the optical center on the sensor perpendicular to the camera’s pinpoint. The parameter *S* is the skew [24].

(1) $$K={\left[\begin{array}{ccc} f_{x} & S & c_{x}\\ 0 & f_{y} & c_{y}\\ 0 & 0 & 1 \end{array}\right]}_{3\times 3}$$

We can calculate the world to image plane transformation with full-rank (4×4) matrices (Equation (2)). Using full-rank matrices allows us to invert them and to calculate the image plane to the world transformation [24].

(2) $$\left[\begin{array}{c} u\\ v\\ 1\\ 1/z \end{array}\right]=\frac{1}{z}{\left[\begin{array}{cc} K & 0\\ 0 & 1 \end{array}\right]}_{4\times 4}{\left[\begin{array}{cc} R & T\\ 0 & 1 \end{array}\right]}_{4\times 4}\left[\begin{array}{c} x_{w}\\ y_{w}\\ z_{w}\\ 1 \end{array}\right]=\frac{1}{z}{\left[\begin{array}{cc} K & 0\\ 0 & 1 \end{array}\right]}_{4\times 4}\left[\begin{array}{c} x_{w}\\ y_{w}\\ z_{w}\\ 1 \end{array}\right]$$

The full-rank transformation matrix ${\left[R|T\right]}_{4\times 4}$ can be omitted, because the skew S=0 and the camera matrix is aligned with the world. The parameters $x_{w}$, $y_{w}$ and $z_{w}$ are the world coordinates for a point in the point cloud. On the camera sensor plane, *u*, *v*, and *z* denote the camera coordinates.

The inverse camera matrix can be analytically calculated and it is defined in Equation (3).

(3) $$K^{-1}=\left[\begin{array}{ccc} 1/f_{x} & -S/(f_{x}f_{y}) & \left(Sc_{y}-c_{x}f_{y}\right)/\left(f_{x}f_{y}\right)\\ 0 & 1/f_{y} & -c_{y}/f_{y}\\ 0 & 0 & 1 \end{array}\right]$$

Then the Kinect image plane to world transformation is defined as:

(4) $$\left[\begin{array}{c} x_{w}\\ y_{w}\\ z_{w}\\ 1 \end{array}\right]=z{\left[\begin{array}{cc} K_{kinect}^{-1} & 0\\ 0 & 1 \end{array}\right]}_{4\times 4}\left[\begin{array}{c} u\\ v\\ 1\\ 1/z \end{array}\right]=z\left[\begin{array}{cccc} 1/f_{x} & 0 & -c_{x}f_{y}/\left(f_{x}f_{y}\right) & 0\\ 0 & 1/f_{y} & -c_{y}/f_{y} & 0\\ 0 & 0 & 1 & 0\\ 0 & 0 & 0 & 1 \end{array}\right]\left[\begin{array}{c} u\\ v\\ 1\\ 1/z \end{array}\right]$$

The projected coordinates from the world coordinates to the hyperspectral camera plane can be calculated with the intrinsic camera parameters $K_{hyper}$ and the extrinsic parameters ${\left[R|T\right]}_{kinect\to hyper}$. The extrinsic matrix defines the transformation between the two camera locations and orientations with the rotation R and the translation T matrices.

The projection matrix of Kinect’s world coordinates to the hyperspectral camera plane is defined in Equation (5).

(5) $$P_{kinect\to hyper}=K_{hyper}{\left[\begin{array}{cc} R_{3\times 3} & T_{3\times 1} \end{array}\right]}_{kinect\to hyper}$$

The world coordinates are then projected on the hyperspectral camera sensor plane:

(6) $${\left[\begin{array}{c} u\\ v\\ 1 \end{array}\right]}_{hyper}=\frac{1}{z_{w}}P_{kinect\to hyper}{\left[\begin{array}{c} x_{w}\\ y_{w}\\ z_{w} \end{array}\right]}_{kinect}$$

Pixels outside of the Kinect’s operational range were filtered out. Hyperspectral image pixel matching is conducted by simply rounding the projected image plane coordinates to nearest even integer pixel coordinates that fit inside the Kinect image plane. The spectral point cloud is defined by ($x_{w}$, $y_{w}$, $z_{w}$) coordinates and their matching 133 spectral bands.

The current point cloud file formats do not support storing more than three color bands. Therefore, we defined a custom format using xarray [25] and netcdf [26] that contains the spatial and spectral information, point normals, and other metadata, such as the band-wise wavelengths. Xarray is a Python library that makes working with multi-dimensional data arrays with different coordinate systems easier. Netcdf is a community standard for sharing array-oriented scientific data.

### 2.6. FPI Hyperspectral Camera to Kinect Calibration

**Figure 5:**
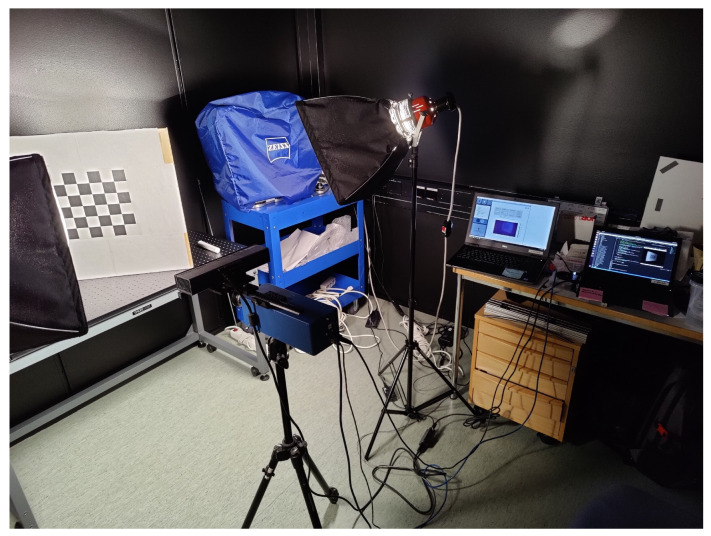
The experimental calibration setup with the hyperspectral camera, Kinect, halogen diffusers, and realignable checkerboard calibration pattern.

The intrinsic and extrinsic camera matrices can be inferred using a common and known reference image pattern. In our case, we used a 9 × 6 checkerboard image with 45 mm square size printed on standard copying paper. We captured 33 calibration images with both cameras while turning the image pattern in different angles along all axis and keeping the camera position fixed. Figure 5 depicts the calibration setup.

The FPI hyperspectral camera was configured to capture four images with different interferometer settings per each calibration image position. That resulted in spectral images with 8 wavelength channels. We produced the final calibration images by clipping the band values within [0,μ+10σ] range to remove any outliers, such as dead pixels, averaging the bands, and normalizing them to gray scale to minimize spatial image noise. We normalized Kinect’s IR images to [0,255] range and used them as-is.

We used OpenCV’s findChessboardCorners function to automatically detect the corners of the checkerboard in the images, cameraCalibrate function to estimate the camera matrix (Equation (1)) parameters for both cameras and stereoCalibrate functions for estimating the extrinsic matrix between the two camera locations and positions with their previously determined optical properties. The resulting intrinsic parameters are listed in Table 1.

### 2.7. Point Cloud Registration

We used Open3D point cloud processing libraries to infer the spatial transformations between point clouds. Our experiment included five point clouds that were captured by moving the hyperspectral camera and Kinect around the target object. The point cloud of the center-most camera position was used as the target for aligning the other point clouds, which will be referred as the source point clouds.

**Figure 6:**
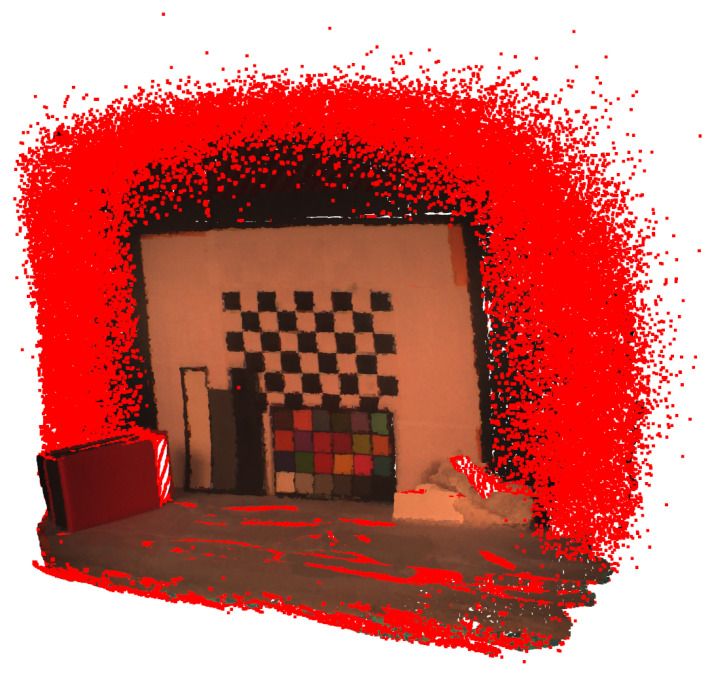
Visualization of the outlier removal for the point cloud in the middle camera viewpoint. The points highlighted with red were removed.

The point clouds had to be preprocessed to filter out excessive noise. Outliers in the point clouds were identified and removed statistically based on the average distance in a neighborhood of points, as shown in Figure 6.

Aligning two point clouds without prior information on their initial pose in space was achieved by using global and local registration algorithms. We computed pose-invariant FPFH features (Fast Point Feature Histograms) [27] which represent the surface model properties around each point. Using FPFH speeds up the global point cloud registration significantly compared to genetic and evolutionary algorithms [27]. We downsampled the point clouds with 15 mm voxel size and estimated the point normals for each point cloud, as FPFH relies on the 3D coordinated and estimated surface normals. Open3D estimates vertex normals by calculating principal axis of the adjacent points over the closest neighboring points.

**Figure 7:**
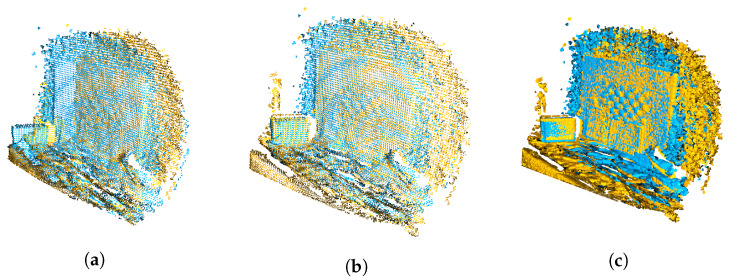
Visualizations of the registration of two point clouds, one shown in blue and one in yellow: (**a**) The point clouds before realignment, (**b**) after global registration, and (**c**) the refined local registration.

Figure 7 illustrates the registration steps. We used RANSAC [28] for the global registration. RANSAC works by picking random points from the source point cloud and finding their corresponding points in the target point cloud by querying the nearest neighbors in the FPFH feature space. A pruning step rejects false matches early. We experimentally set RANSAC pruning algorithm’s correspondence distance threshold (the distance between two aligned point) to 75 mm. The algorithm’s correspondence edge length was set to 0.9. It is a threshold for checking that any two arbitrary corresponding edges (line between two vertices) in the source and target point clouds are similar. The RANSAC convergence criteria was set to 400,000 iterations and 0.999 confidence.

**Figure 8:**
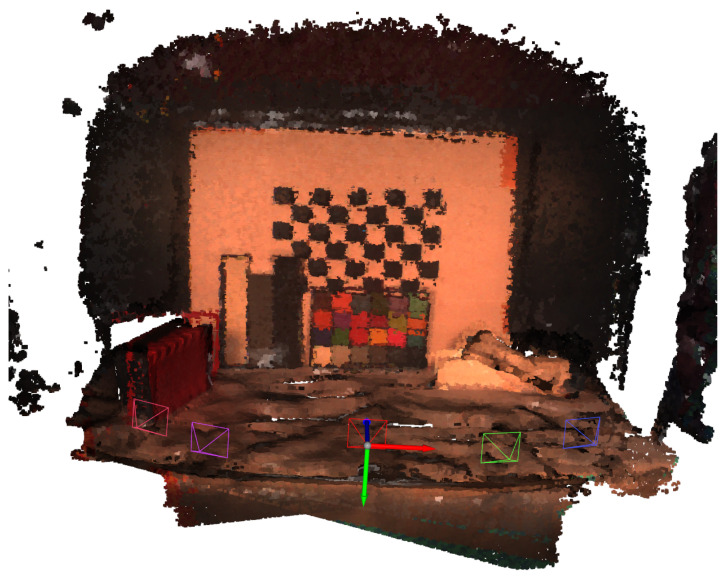
The fully registered point cloud with pseudo RGB coloring and the visualizations of camera capture viewpoints.

The next step is the local refinement with the point-to-plane ICP (Iterative Closest Point) [29] registration algorithm. We used the original outlier-filtered point clouds without downsampling and the rough transformation results from RANSAC to further refine the alignment. Figure 8 shows the fully registered point cloud with pseudo coloring and the camera viewpoints.

ICP produces the extrinsic transformation matrices to integrate each point cloud to the viewpoint of the central camera position. The transformation matrices also gives us the positions and orientations of each camera in relation to the central camera. We can use them later to calculate the emission angles.

### 2.8. Calculating Point Normals

**Figure 9:**
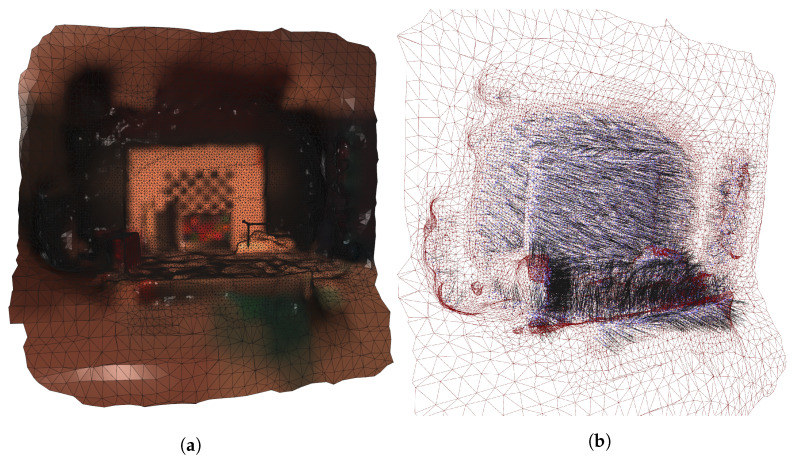
(**a**) The fitted mesh on the fully registered point cloud with Taubin smoothing. (**b**) Visualization of the mesh triangles (red) and the points point cloud (purple) with the recalculated surface normals (black).

The next step is to calculate corresponding point normals for each point in the point cloud. Normals are needed to calculate the emission angles relative to the camera positions. We used Open3D’s Poisson surface reconstruction method [30] to fit a surface on the point cloud. Open3D offers functions to calculate point normals using its adjacent points. The reconstruction algorithm allows defining the depth of the underlying octree data structure. It controls the resolution of the resulting triangle mesh. We set the depth to 7, because the noise could create steep angles on flat surfaces. We applied Taubin smoothing [31] to further smoothen the fitted surface. The produced mesh is presented in Figure 9a.

We assigned the normals of the closest mesh triangles to the points of the point cloud using Open3D’s ray casting functions. Figure 9b illustrates a downsampled view of the new point normals showing how flat surfaces have relatively uniform normal directions.

### 2.9. Calculating Emission Angles

**Figure 10:**
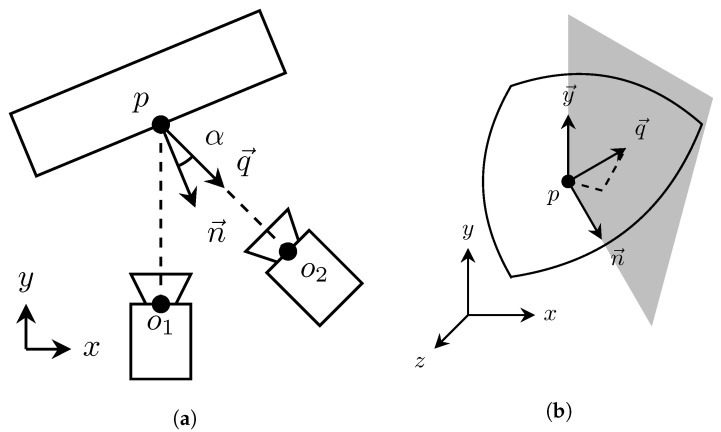
(**a**) Emission angle calculation for the point *p*. (**b**) Illustration of the vertical plane that splits the space along the direction of the point normal and the y axis. It is used for determining the sign of the emission angle.

The emission angle α is the angle at which the reflected and transmitted light are received at the detector. Defining the emission angle at the surface point *p* then comes down to calculating the cosine between the surface normal and the vector at the direction of camera from the point *p*, as illustrated in Figure 10a.

The relative camera position $\vec{o}$ is acquired from the world camera translation we estimated during the registration. The translation vector *T* needs to be negated, because the original world-to-world transformations are defined towards the origin, the middle camera:

(7) $$\vec{o}=\left(-T_{x},-T_{y},-T_{z}\right)$$

The emission angle α at the point $\vec{p}$ can be calculated as the dot product of the normal vector $\vec{n}$ and the vector $\vec{q}$ pointing from the point $\vec{p}$ towards the capturing camera position $\vec{o}$:

(8) $$\vec{q}=\vec{o}-\vec{p}$$

(9) $$\alpha =\cos^{-1}\left(\frac{\vec{n}\cdot \vec{q}}{∥\vec{n}∥∥\vec{q}∥}\right)$$

The resulting emission angle is defined in [0,90] degree range. Our camera is only moved in xz-axis and the position in y axis is kept relatively fixed with approximately 2 cm variation between capture positions. Therefore, we calculate the signs of the emission angles to have [−90,90] degree range. We split the space with vertical plane along the direction of the point normal $\vec{n}$ and the y axis.

(10) $$\alpha_{signed}=\alpha \mathrm{sign}\left(\left(\vec{n}\times \vec{y}\right)\cdot \vec{q}\right)$$

The sign of the dot product between the splitting plane and camera pointing vector $\vec{q}$ determines on which side of the plane point $\vec{p}$ is located. Figure 10b depicts the splitting plane and the related vectors. Equation (10) gives us the signed emission angle.

### 2.10. Reflectance and Its Angular Dependence

Reflectance is defined as a material’s ability to reflect incoming electromagnetic radiation. Reflectance is a unitless quantity between zero and one; a material with reflectance of one will reflect all radiation incident on it, and a material with reflectance of zero will not reflect anything. In this work, the quantity of interest is spectral reflectance, a set of reflectances each corresponding to a wavelength channel. In addition to wavelength, reflectance can depend on the directions of incident and reflected light [32].

Reflection can be divided into specular reflection from an optically smooth surface, such as a mirror, and diffuse reflection from a rough surface such as soil. Reflections from real surfaces are often a mix of these two. For example, a body of water will reflect the image of a light source in one direction, and in another direction appear the color of the solids suspended in the water [32].

The simplest analytical expression for reflection from diffuse surfaces is known as Lambert’s law. The law is based on the observation that the apparent brightness of a surface is independent of the angle it is viewed from. Lambert’s law states that the only directional dependence to the intensity of reflected light comes from the incidence angle, as this affects the intensity of incident light. Although the reflections of real surfaces are not perfectly Lambertian, some bright surfaces come close [32].

To find the spectral reflectance of a surface, one must quantify both the light reflected from the surface and the light arriving to it. This is often done by measuring the unknown surface along with a standard that has known reflectance properties. If the standard is assumed to be perfectly white, i.e., it reflects all light arriving to it in the wavelength region of the measurement, the spectral reflectance *R* is given by:

(11) $$R=\frac{I}{I_{white}},$$

where *I* is the spectral radiance reflected from the target, and $I_{white}$ is the spectral radiance reflected from the white reference target [32]. A similar approach was taken to calculate spectral reflectance from our measurements. The used hyperspectral camera recorded a spectral radiance *I* for each of its pixels. A white reference measurement was made by placing a block of Spectralon [33] reference material in the imaged scene. Spectralon is a common reflectance standard that is highly reflecting and diffuse in our spectral range extending from visual wavelengths to the shorter end of near-infrared. The reference radiance $I_{white}$ was calculated by averaging the spectral radiance over the reference target area.

The lamps used to illuminate the scene were positioned and aligned on both sides of the camera so that the specular reflections were minimized to the front view of the color checker board. With this lighting geometry, the measured reflectances should show higher values when the target was imaged at a side view. The target is expected to have a specular reflection component in its reflection.

## 3. Results

**Figure 11:**
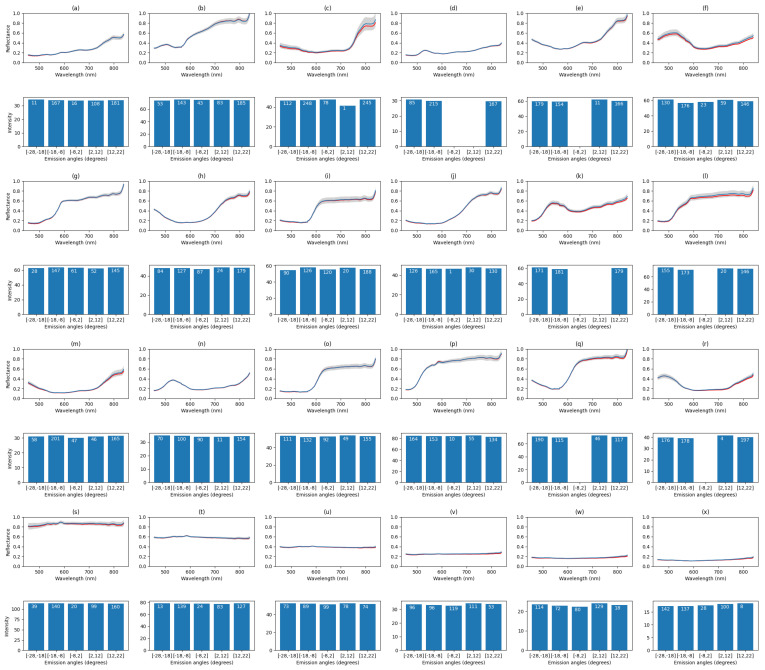
The average spectra and the intensity histograms of each matching color tile (**a**–**x**) of the reference color checker in Figure 12. The average spectra of all measurements are plotted on top in blue, the front view average spectrum is plotted in red and the intensity histograms, the band-wise sum of the reflectances, per emission angle are on the bottom. The sample count per a histogram bin is displayed in white. The gray color in the top plots illustrate the band-wise standard deviations.

Figure 11 depicts the measurement results for each tile in the color checker. Each tile has been measured with the experimental setup from five different angles and their average spectra were plotted in the top and the intensity histograms, the band-wise sum of the reflectances, per emission angle on bottom, respectively. Each tile was cropped by hand from the fully registered point cloud.

**Figure 12:**
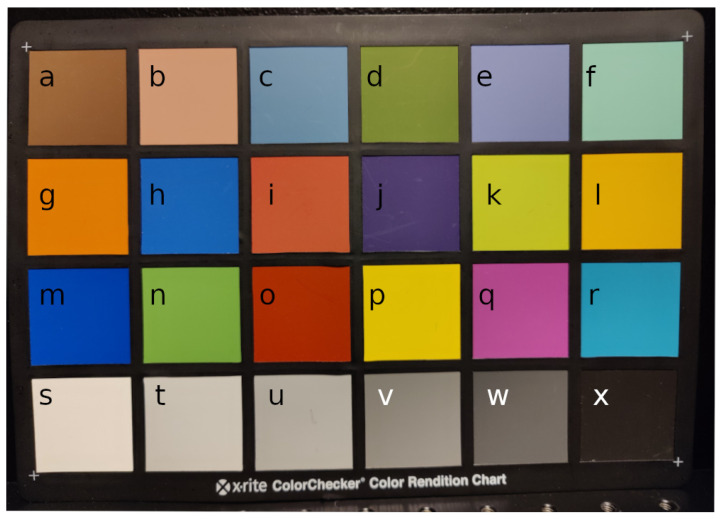
The color checker board used for calculating the result reflectances. Letters a–x correspond to each tile and a result plot in Figure 11.

The tiles on the color checker board in Figure 12 correspond to the plots in Figure 11. The measured emission angles on the checker board varied between −28 and 22 degrees. The results verify that the color checker tiles are mostly diffuse surfaces and the emission angle has little effect to the intensity. Some liminal intensity attenuation can be observed around zero emission angle. This is expected as the positioning of the lamps cause the front view have dimmer illumination. The side views receive more light due to specular reflections.

In Figure 11, the red plots illustrate the average measured spectrum from the front view. It represents the spectrum of the original hyperspectral image without emission angle information. The blue plot is the average over all measurements in different angles. The gray color in the plots illustrates the band-wise standard deviation. Some color tiles showed larger band-wise intensity fluctuations. More fluctuations are observed at the last bands. This may due to the fact that this type of camera has previously been observed to produce noise in large wavelengths. However, we can see more deviance depending on the tile color. For example, the most reflective tile *s* (white) shows more noise than the darker toned tiles *t*–*x*. In tiles *c*, *f*, and *r* we noticed fluctuations in the smaller wavelengths, and for *j*, *l*, *o*, and *p* in the middle wavelengths. Common for all the noisiest wavebands is that they all share large intensity values. The red plots shows that for most of the colors, the spectrum does not change much between different views, as could be seen from the intensity histograms as well. The results suggest that the spectra from different angles are similar as in the original front view hyperspectral image.

In Table 2, we list the RMSE (root mean squared error) values and their standard deviations for each tile. The errors are calculated between the mean spectra of the central camera view and the spectra from other view positions. The point of this measurement is to quantify how much the emission angle affects the measured spectra. The deviation values ranged from approximately 0.01 to 0.05, which can be regarded as small, as could be stated based on the intensity histograms. We used cosine, sometimes called the spectral angle [34] in the spectral domain, to measure the differences in spectrum shape. The differences are between 1.5 and 3.2 degrees, which would refer to emission angle having little effect on the shape of the spectrum with these targets.

We kept the lighting setup fixed and, as seen from the results, we observed only small fluctuations in the intensity values while moving the camera. Based on a visual inspection the colorchecker board appeared diffuse, and, as such, could be approximated as Lambertian. The intensity of light reflected from Lambertian surfaces is not dependent on the emission angle and, thus, we would expect to see no variation in intensities measured from different angles. The results of the experiment are in line with this expectation.

In Table 3, we list registration errors of the individual point clouds that were used for creating the final dense point cloud. The correspondence set size is the number of point pairs that have correspondence to each each other in the source and target point clouds. The fitness is the number of inlier correspondences divided by the number of points in the target point cloud. A larger value is better and it means that the point clouds have more overlap. The RMSE (root mean squared error) is calculated over all the inlier correspondences. Each pruned point cloud had approximately 140,000 points. The quite high correspondence set size (approx. 40,000–50,000) and fitness (0.29–0.36) values suggest that the registered point clouds had a lot of overlap which made the resulting registration quite reliable as we can visually confirm on the resulting full point cloud in Figure 8. However, we can see some off-alignment on the checkerboard.

## 4. Discussion

Figure 8 illustrates the final point cloud with pseudo coloring. Due to inaccuracies in the Kinect-to-FPI hyperspectral camera calibration, some transformations are off by 1–2 cm at most. The most significant error source was the sub-optimal mounting of Kinect. Small deviations in the orientation between the two cameras caused large errors in extrinsic parameter calculations, as the camera had to be moved to capture images on multiple angles.

Originally, the hyperspectral camera used optics that had a small field of view, for which OpenCV could not satisfactorily solve the extrinsic parameters. Therefore, we selected lens that matches closely the field of view of Kinect. The calibration would have benefited from averaging multiple infra-red pictures from the Kinect to reduce noice.

We observed that the point clouds captured by Kinect gauged depth values depending on the brightness of the target, darker areas gaining shorter distances than the brighter areas. This is especially visible in the checkerboard pattern in Figure 7c. The intensity related error is known to occur with Kinect V2 [35]. We fitted a mesh on the plane and recalculated the point normals to diminish these alterations to the emission angles of the spectra. One future improvement would be matching the point cloud normals by ray tracing and finding the intersecting mesh triangles.

Looking at Figure 11, we see a spike in the average spectra around infrared range. This is expected as the Kinect V2 illuminates the scene and it is detected by the hyperspectral camera. This should be taken into account when using this kind of time-of-flight depth camera, if the application operates around these wavelengths.

To incorporate more accurate and dense spectral point cloud, using the rest of the spectra in hyperspectral data cubes should be considered. In the presented implementation, only the closest spatially matching spectra in the Kinect’s perspective were considered and the rest were pruned.

A previous study by [14] showed how linescanner hyperspectral camera and Kinect V2 can be used to create a 3D white referencing library which offers tilt angle specific white references for hyperspectral calibration. The motivation of the study was to improve the calibration of soy bean leaf images. The authors used a ball shaped white reference in creating the 3D white referencing library, which is something we should consider using in our emission angle dependent reflectance measurements. The authors detected a significant difference at the angled reflectance calibration compared to a flat reference. We used an averaged white reference spectrum from a flat reference object over multiple angles. In the study, the authors envisioned using LiDAR sensors in 3D scanning and use it in field environments. The strength of LiDAR is in long distance applications. The presented method should work in mid-range complex surface imaging applications and be more portable in comparison.

Future research topics would include fitting a model, such as a neural network, with the data produced by the system and use it to interpolate the emission angle dependent reflectance of glossier materials. Using a lighting setup, such as the one presented in this research, we could potentially infer the reflectance at an emission angle where specular reflections are minimized. Specular reflections occur on glossy surfaces.

Exchanging the two light sources of the experimental setup for just one with a collimated output and tracking the relative position of the camera system and the light source would allow inferring the incidence angle of light from the 3D point cloud. With both incidence and emission angles and spectral data recorded for each pixel of the hyperspectral image, the method could be used to measure a spectral version of the bidirectional reflectance distribution function (BRDF). BRDF is a reflectance quantity that is related to the changes in reflectance with different incidence and emission angles of light. BRDF can be used for material characterization and, for example, producing digital textures. Typically measurements of this quantity for a sample require maneuvering either a light source and a detector [36] or the sample itself [37] to accurately to measure it with a series of incidence and emission angles. With the setup described in this study, including the light source upgrade, one could determine the BRDF of a material with very few measurements taken of a rounded sample. The curved surface would include a wide array of incidence and emission angles, possibly enough to construct a spectral BRDF for a material from just one capture.

## 5. Conclusions

We demonstrated a sensor fusion method for combining data from frame-based hyperspectral and a depth camera. We created an experimental application on how to utilize the depth augmented hyperspectral data to measure emission angle dependent reflectance from a multi-view inferred point cloud.

The method could successfully combine the 3D point cloud data and hyperspectral data from different viewpoints. The calculated angle dependent reflectance results refer that the target color checker board has Lambertian surface properties. The significance of this study is in the remarks and implementation details of designing a system for an imaging application augmenting frame-based hyperspectral data with time-of-flight depth camera data, as well as in the future research ideas we presented in the discussion chapter.

## Figures and Tables

**Table 1:** The estimated intrinsic parameters of the cameras during the calibration.

Intrinsic Parameter	Kinect V2	FPI Hyperspectral Camera
$f_{x}$	366.261	1382.955
$f_{y}$	366.465	1383.227
$c_{x}$	255.923	1002.023
$c_{y}$	206.977	601.358

**Table 2:** Root mean squared differences and spectral angles (cosine) of measured spectra for each corresponding color checker tile, in Figure 12, compared to the averaged front view spectrum from central camera position.

	a	b	c	d	e	f	g	h	i	j	k	l
**RMSE**	0.0248	0.0427	0.0493	0.0125	0.0301	0.0339	0.0273	0.0219	0.0474	0.0222	0.0316	0.0413
std	0.0039	0.0093	0.0136	0.0004	0.0050	0.0069	0.0039	0.0029	0.0133	0.0028	0.0065	0.0105
**cos**	2.4171	1.5101	2.4386	2.1830	1.7203	1.7153	1.5530	1.7175	1.7402	1.5635	1.6439	1.4640
std	1.5109	0.8921	1.8757	0.4083	0.9819	1.2294	0.7569	0.8792	1.8425	0.5303	1.1196	0.8974
	**m**	**n**	**o**	**p**	**q**	**r**	**s**	**t**	**u**	**v**	**w**	**x**
**RMSE**	0.0259	0.0121	0.0397	0.0416	0.0313	0.0311	0.0338	0.0200	0.0121	0.0145	0.0108	0.0096
std	0.0035	0.0011	0.0097	0.0081	0.0067	0.0058	0.0047	0.0016	0.0003	0.0008	0.0003	0.0006
**cos**	2.8011	1.9333	1.6055	1.4051	1.3058	2.4644	1.2502	1.3298	1.5481	1.9872	2.5215	3.1784
std	2.4471	0.6890	1.2743	0.9680	0.6200	2.2738	0.8716	0.5919	0.2548	0.3869	0.4246	0.9830

**Table 3:** The refined local registration errors of the listed source point clouds (side views) to the target camera position (center).

Viewpoint	Fitness	Inlier RMSE	Correspondence Set Size
Left	0.356	1.975	50,192
Left-most	0.296	1.989	41,620
Right	0.358	2.005	49,098
Right-most	0.2901	2.002	40,006

## Data Availability

The data presented in this study are openly available in Zenodo at [https://doi.org/10.5281/zenodo.7108216, accessed on 1 October 2022].

## Abbreviations

The following abbreviations are used in this manuscript:

BRDF	Bidirectionald reflectance distribution function
DOF	Depth of field
FOV	Field of view
FPFH	Fast point feature histogram
FPI	Fabry–Pérot interferometer
HSI	Hyperspectral Imaging
RANSAC	Random sample and consensus
WD	Working distance
